# Impact of Right-Sided Nephrectomy on Long-Term Outcomes in Retroperitoneoscopic Live Donor Nephrectomy at Single Center

**DOI:** 10.1155/2013/546373

**Published:** 2013-10-21

**Authors:** Kazuya Omoto, Taiji Nozaki, Masashi Inui, Tomokazu Shimizu, Toshihito Hirai, Yugo Sawada, Hideki Ishida, Kazunari Tanabe

**Affiliations:** Department of Urology, Tokyo Women's Medical University, 8-1 Kawada-cho, Shinjuku-ku, Tokyo 162-8666, Japan

## Abstract

*Objective.* To assess the long-term graft survival of right-sided retroperitoneoscopic live donor nephrectomy (RPLDN), we compared the outcomes of right- and left-sided RPLDN. *Methods.* Five hundred and thirty-three patients underwent live donor renal transplantation with allografts procured by RPLDN from July 2001 to August 2010 at our institute. Of these, 24 (4.5%) cases were selected for right-sided RPLDN (R-RPLDN) according to our criteria for donor kidney selection. Study variables included peri- and postoperative clinical data. *Results.* No significant differences were found in the recipients' postoperative graft function and incidence of slow graft function. Despite significant increased warm ischemic time (WIT: mean 5.9 min versus 4.7 min, *P* < 0.001) in R-RPLDN compared to that in L-RPLDN, there was no significant difference between the two groups regarding long-term patient and graft survival. The complication rate in R-RPLDN was not significantly different compared to that in L-RPLDN (17% versus 6.5%, *P* = 0.132). No renal vein thrombosis was experienced in either groups. *Conclusions.* Although our study was retrospective and there was only a small number of R-RPLDN patients, R-RPLDN could be an option for laparoscopic live donor nephrectomy because of similar results, with the sole exception of WIT, in L-RPLDN, and its excellent long-term graft outcomes.

## 1. Introduction

The first laparoscopic living-donor nephrectomy (LLDN) was performed by Ratner et al. [1]. Since then, there has been increased acceptance of the procedure in many countries. In Japan, the number of deceased donor kidneys available for transplantation has not shown any increase, but living donor kidney transplantation increases yearly [2]. Laparoscopic procurement can offer an advantage to living kidney donors. The recent outcome of laparoscopic donor nephrectomy seems to be greatly improved compared to that in the early years, but the procedure still remains challenging even for the most experienced laparoscopists [2–4]. This is apparent especially in contrast to open donor nephrectomy, in which the right kidney was removed in 24% to 35% of the patients. The rate of right-sided donor nephrectomy in various laparoscopic series ranges from 3.5% to 56.2% [5, 6]. According to the database from the United Network of Organ Sharing (UNOS), right LLDN represented only 10.5% of all LLDN in 2006 [7]. One reason for the reluctance to perform right-sided laparoscopic donor nephrectomy has been the high vascular complication rate and the technical difficulties reported in the initial series. Moreover, right-sided laparoscopic donor nephrectomy is associated with a small increased risk of allograft failure [7].

The most common laparoscopic approach is the transperitoneal approach as it provides adequate working space and easy dissection. However, in comparison with LLDN, retroperitoneoscopic live donor nephrectomy (RPLDN) necessitates a direct and quick approach to the vessels in the renal hilum, without interference to the liver, spleen, pancreas, or bowel. On the other hand, RPLDN is used by just a few institutes worldwide, and there have been only a few reports with regard to studying the comparison between right- and left-sided RPLDN (L-RPLDN), which include using a hand-assisted technique [5, 8, 9]. We have been trying since 2001 to establish the technique of RPLDN without using hand assistance [10, 11]. We retrospectively reviewed our single center experience of right-sided RPLDN (R-RPLDN) in order to evaluate the efficacy and safety, especially with regard to vascular complications and long-term graft survival rates. 

## 2. Materials and Methods

Between July 2001 and August 2010, 533 consecutive procedures of RPLDN were performed at our institute. Of these, 24 (4.5%) cases were R-RPLDN. In order to obtain the longer renal vein and to avoid thrombosis at the renal vein, we principally selected L-RPLDN, even if the left kidney had multiple renal arteries. However, the kidneys with more than three arteries were avoided for donation, since the reconstruction of such arteries is seriously complicated and difficult. We also selected the right kidney which had anatomical or functional problems (see Table 1). After using our criteria for donor kidney selection, the final number of R-RPLDN in our study was extremely small. 

 Donor preoperative parameters analyzed included operating time, time to procurement of the kidney, estimated blood loss, warm ischemic time (WIT), total ischemic time (TIT), CO_2_ gas pressure, and days of hospital stay. WIT indicates the time from clamping of renal artery to flushing of the kidney with cold solution. Assessment of recipients' outcomes included analysis of serum creatinine levels in slow graft function (SGF; serum creatinine level is more than 3.0 mg/dL at 4 days after transplantation), delayed graft function (DGF; patients required hemodialysis after transplantation due to acute tubular necrosis.), acute rejection rate, and long-term patient and graft survival rate. These data were collected retrospectively using hospital charts. Statistical analyses were performed using the Mann-Whitney *U*-test for individual variables, Fisher's exact test for categorical data, and the log-rank test for patient and graft survival rates.

### 2.1. The Surgical Procedure for RPLDN

**Figure 1:**
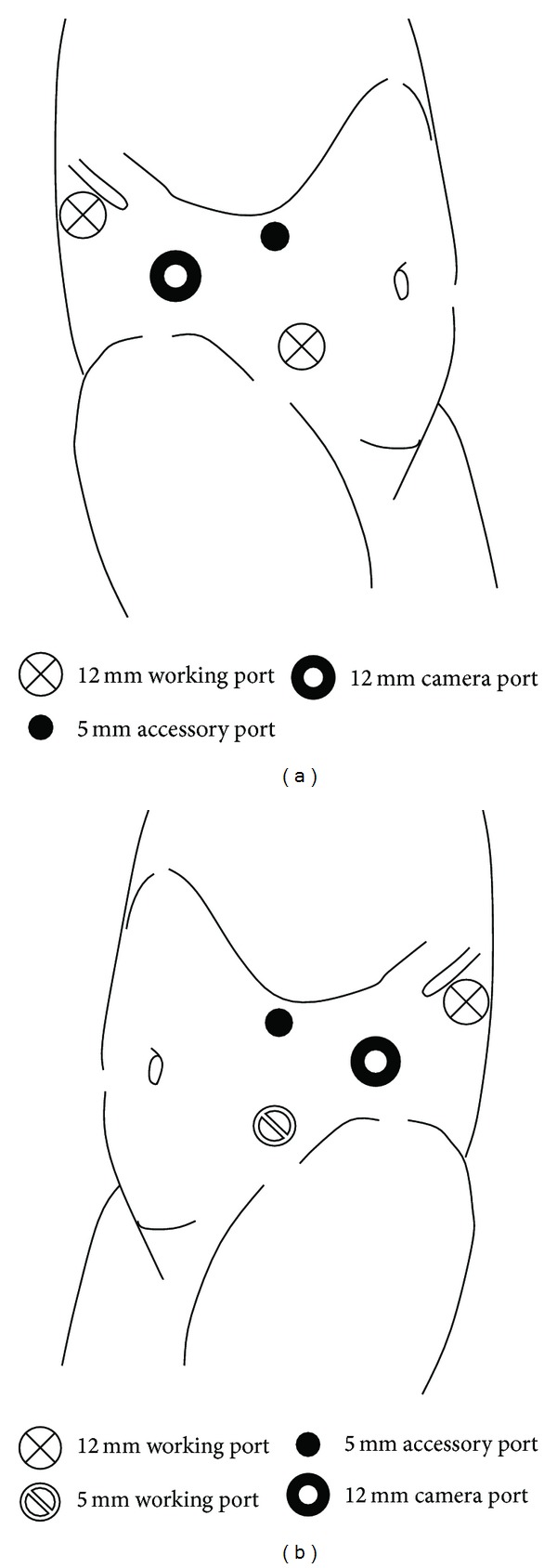
Port placement and pfannenstiel incision in right and left RPLDN.

We described in detail the technique for surgical procedure for RPLDN in our recent report [10]. Three retroperitoneoscopic ports were inserted in the initial cases. Recent cases were performed using four ports (Figure 1). The retroperitoneal space was insufflated to a pressure of 7–10 mmHg. The hand-assisted technique was not used in any of the cases. The renal artery and vein were severed sequentially using Endo-GIA staplers. The kidney was placed in the bag and extracted though the Pfannenstiel incision.

### 2.2. Recipient Surgery

The standard renal transplant technique was employed in all patients. All surgery on recipients with right renal allografts was performed by severing the internal iliac vein to obtain high mobility of the external iliac vein. However, in one recipient having a right renal allograft it was needed to extend the renal vein using the saphenous vein in the recipient. The ureter was implanted into the bladder using the Lich-Gregoir technique without stenting.

## 3. Results

Characteristic demographics for donors and recipients in R- and L-RPLDN are shown in Table 2. Donor nephrectomy was performed successfully in all patients. There were no significant differences between right and L-RPLDN with regard to donor age, sex, and mean body mass index. Four of 24 (17%) in the R-RPLDN group and 123 of 509 (24%) in the L-RPLDN group had multiple renal arteries (*P* = 0.550). On the one hand, six of 24 (25%) in R-RPLDN and 31 of 509 (6%) in L-RPLDN had multiple renal veins, which indicates a significant difference between R- and L-RPLDN (*P* = 0.002). They were also significantly different regarding the average length of the renal arteries (4.7 ± 0.9 versus 3.7 ± 0.7 cm; *P* < 0.001) and veins (1.8 ± 0.6 versus 4.0 ± 0.8 cm; *P* < 0.001) between R- and L-RPLDN. In recipients, there was no significant difference regarding recipient age, sex, and immunosuppressive regimens between R- and L-RPLDN. Also there was no significant difference in the immunological background of recipients such as the incidence of HLA-identical, or ABO-incompatibility and preoperative donor-specific HLA antibody between R- and L-RPLDN. 

Postoperative outcomes of R- and L-RPLDN are shown in Table 3. The operating time was 326 ± 67 minutes in R-RPLDN and 312 ± 71 minutes in L-RPLDN (*P* = 0.482) The time to procurement of the kidney was 265 ± 66 and 269 ± 66 minutes in R- and L-RPLDN, respectively (*P* = 0.407). The estimated blood loss was 61 ± 59 and 51 ± 60 g in R- and L-RPLDN, respectively (*P* = 0.218). The postoperative stay was 4.1 ± 1.2 and 3.7 ± 1.4 days in R- and L-RPLDN, respectively (*P* = 0.245). The TIT was not significantly different between right (104 ± 27 minutes) and L-RPLDN (100 ± 31 minutes; *P* = 0.158). However, the WIT was significantly different between R- (5.9 ± 1.9 minutes) and L-RPLDN (4.8 ± 1.5 minutes; *P* < 0.001). Two of 24 cases (8.3%) in R-RPLDN required more than 10 min in WIT (10 and 11 min, resp.). Also the average CO_2_ gas pressure during the procedure was significantly different between R- (7.8 ± 1.2 mmHg) and L-RPLDN (7.2 ± 1.3 mmHg; *P* = 0.038). In early graft function, no patients with a transplanted kidney from R- or L-RPLDN required hemodialysis after transplantation due to acute tubular necrosis. Also there was no significant difference between R- and L-RPLDN in regard to the incidence rate of SGF that was defined as a serum creatinine level of more than 3.0 mg/dL at 4 days after surgery without rejection (0% versus 2.0%; *P* = 0.838). Although the acute rejection rate in R-RPLDN (21%) was higher than that in L-RPLDN (12%), there was no significant difference between them (*P* = 0.315) due to small number in R-RPLDN. The serum creatinine level at 1 day after transplantation in R-RPLDN was significantly higher than that in L-RPLDN (4.5 ± 2.4 versus 3.7 ± 1.9 mg/dL; *P* = 0.038). However, there was no significant difference regarding the serum creatinine level at seven days (1.8 ± 1.7 versus 1.4 ± 1.0 mg/dL; *P* = 0.097) and 14 days (1.6 ± 1.7 versus 1.5 ± 3.4 mg/dL; *P* = 0.957) between R- and L-RPLDN. In long-term outcomes, there was no significant difference regarding the patient and graft survival rate between R- and L-RPLDN. The seven-year patient survival rate using R- and L-RPLDN is 100% and 97.8%, respectively. Also the seven-year graft survival rate using right and left RPLDN is 92.3% and 90.4%, respectively.

Complications of R-RPLDN are shown in Table 4. In all cases, no vascular thrombosis occurred. Four of 24 (17%) cases in R-RPLDN experienced wound infection, postoperative hemorrhage without blood transfusion, subcutaneous emphysema, and ureter reconstruction. We did not experience any cases with SGF in R-RPLDN. The overall complication rate (17%) in R-RPLDN is higher than that in L-RPLDN (6.5%), although there was no significant difference between them (*P* = 0.132). One donor in L-RPLDN was converted to open nephrectomy because of severe adhesions around the renal vein due to a previous lymphadenectomy for ovarian cancer, which was curable, and potential transmission of the cancer could reasonably be excluded because there had been no recurrence of the cancer for more than ten years. No serious complications, such as massive bleeding or bowel injury, were encountered. Seven cases of 533 (1.3%) experienced postoperative hemorrhage. Flank incision was added in one donor for hemostasis after L-RPLDN, and four donors had blood transfusion alone. One case in the L-RPLDN group experienced pulmonary embolism after surgery that was successfully treated without any complications. None of the donors required readmission.

## 4. Discussion

Although LLDN is the gold standard method for living kidney donation, right-sided LLDN (R-LLDN) has been associated with short length of the renal vein and venous thrombosis in the recipients [12]. For this reason, many institutes refrain from laparoscopically procuring right-side kidneys for transplantation even if multiple renal arteries are present on the left side [13]. Other authors have reported an increased risk of liver damage from retraction in R-LLDN [14]. According to these backgrounds and reports, the percentage of right kidney procurement is lower than 10% even in various institutes which have considerable experience of LLDN [2, 7, 15, 16]. On the other hand, it has been reported that R-LLDN is faster and safer than left-sided LLDN (L-LLDN) and does not adversely affect graft function and that R-LLDN may be advocated to allow donors to benefit from the advantages of laparoscopic surgery [17]. The reason that R-LLDN is faster and safer than L-LLDN is the anatomic position, which is more caudal in the abdomen and overlying the right flexure of the colon so that it is more easily mobilized than the left flexure, which is attributed to shorter operation times for R-LLDN. Moreover, the venous anatomy is simpler at the right side where there is no need to dissect branches of the renal vein. Ko et al. published a retrospective study of 41 R-LLDNs of 400 total LLDNs performed between 1999 and 2007 and reported similar vascular complication rates. At two years, a followup of right and left kidney graft function in this series showed that they were similar in outcome [18]. Finally, to assess the superiority of R-LLDN over L-LLDN, a randomized trial would be needed.

With regard to RPLDN, Gill et al. published a report on the retroperitoneoscopic approach to donor nephrectomy in which the successful allograft outcome was achieved without vascular complications [19]. The retroperitoneal approach provides two major advantages. First, it offers a direct and quick access to the blood vessels compared to the transperitoneal approach. Second, it does not interfere with any abdominal organs such as the spleen, liver, pancreas, or bowel. The necessity of mobilizing the colon or liver in transperitoneal donor nephrectomy is obviated. However, there are only a few reports on R-RPLDN without using a hand-assisted technique [5, 6, 8, 20] since the RPLDN is used by only a few institutes worldwide. In a contemporary series on RPLDN, the right kidney was removed in 24% to 9.4% (average 18 ± 7.6%) of cases [5, 8, 21]. In our study, 24 cases of 533 (4.5%) were performed by R-RPLDN in which the percentage of right kidney procurement was lower than that in the other institutes. We preferably chose the left kidney in RPLDN because of the longer left renal vein, which facilitates the anastomosis process and to avoid venous thrombosis. Even if the left kidney had multiple renal arteries, we would still choose the left kidney because of the early graft function, except where there were three renal arteries, and have found that the outcome is similar to that where there is a single renal artery (data not shown). There is a consensus that the “better” kidney should always remain with the donor, so that in case of various conditions, such as vascular renal artery aneurysm and stenosis, large cyst, renal stone, inferior renal function, or anomaly vessels, we would procure the right kidney.

 The WIT with R-RPLDN was significantly longer than that with L-RPLDN. There are two reasons for longer WIT with R-RPLDN. First, it can take more time to carefully sever and secure the right renal vein using staplers in order to obtain a relatively longer renal vein. Second, with regard to the number of renal veins, the incidence of multiple renal veins in R-RPLDN was statistically significant higher than that in L-RPLDN (25 versus 6.1%; *P* = 0.002).

In our study, renal vein thrombus has not been experienced. We prefer to use an Endo-GIA stapler, but not an Endo-TA 30 stapler on both the renal artery and vein. Even when using the Endo-GIA stapler, the average length of the right renal vein is 1.8 cm, which is not too short. In order to prevent vascular complications, as much as possible, we severed the internal iliac vein to obtain high mobility of the external iliac vein and to anastomose easily to the right renal vein. However, in one of 24 (4.2%) recipients it was necessary to use the right renal allograft to extend the renal vein using the saphenous vein since the right renal vein was too short (the length was only 5 mm).

Recently, Bachmann et al. reported that RPLDN is comparable to the open approach with respect to operating time, WIT, and the overall complication rate [22]. And Ruszat et al. also demonstrated that compared to the pure LLDN and hand-assisted LLDN, they experienced significant lower operating times and WIT with RPLDN [23]. The operating time and WIT in our study is certainly longer compared to that of these reports. There are a few reasons for this. Most of our recent cases have been performed by trainees under direct guidance by an experienced laparoscopic surgeon. In difficult cases, such as those with severe adhesion or complex vessels, the operator was changed from the trainee to the laparoscopic mentor. The RPLDN technique requires a long learning curve. In addition, there continues to be a constant influx of new residents and fellows who are exposed to this technique at our academic teaching institution so we often provide them with an invaluable intraoperative teaching experience. On the other hand, the skillful laparoscopic mentor can procure most kidneys within 2 hours, which indicates the operating time is around 150 min (data not shown).

Despite the longer operating time and WIT, we have not experienced SGF and DGF in right RPLDN. Although the serum creatinine level at one day after transplantation in R-RPLDN is significantly higher than that in L-RPLDN, which might be contribute to a longer WIT on R-RPLDN, there is no significant difference between them in regard to the serum creatinine level at seven or 14 days after transplantation. Lee et al. demonstrated that both SGF and the presence of acute rejection have a negative impact on the long-term patient and graft survival rates [24]. In our study, although the incidence rate of acute rejection in R-RPLDN is slightly higher than that in L-RPLDN (21% versus 12%; *P* = 0.315), the SGF was not found in R-RPLDN. In the long-term outcome, the seven-year patient and graft survival rate of R-RPLDN is similar to that of L-RPLDN (100% versus 97.8%; 92.3% versus 90.4%, resp.). 

With regard to complications of R-RPLDN, four cases of 24 (17%) did experience some. Other researchers have reported that their total complication rate was 32% (9 cases of 28) in R-RPLDN [8]. No serious complications, such as massive bleeding, rhabdomyolysis or bowel injury, were encountered in R-RPLDN. Ureter reconstruction which was caused by stenosis has been experienced in our R-RPLDN. This right-side kidney had 8 × 9 mm of renal artery aneurysm, which it was necessary to remove and to reconstruct the vessels on the back table. The specific blood supply to the ureter might have been damaged during dissection of the aneurysm and reconstruction of the renal arteries. In general, one of the indications of R-RPLDN is that the right kidney has abnormal vessels such as renal arteries with stenosis or aneurysm, so we should be very careful to treat them in a manner to avoid injury of the blood supply to the ureter. The absolute number of patients was small and therefore a conclusion with regard to complications can not be described.

In the United States in 2005, pure laparoscopy was favored over the hand-assisted technique [25]. However, the hand-assisted technique is superior to the laparoscopic technique regarding operating time [26]. Recently, the transplant institutes, which are mainly performing the hand-assisted technique for live donor nephrectomy, have been gradually increasing. In order to shorten the operating time, the hand-assisted technique may be selected, especially in living donors with a high level of body mass index (BMI). It is considered that a longer operating time may cause serious complications such as rhabdomyolysis. Fortunately, we have never experienced rhabdomyolysis despite a long operating time, which indicates that an almost normal range of BMI may also contribute to a low incidence rate of severe complications.

In conclusion, our R-RPLDN is an acceptable technique for donor nephrectomy because of having a similar outcome to L-RPLDN and provides excellent graft function after transplantation. Although our study was retrospective and there were only a small number of R-RPLDN patients, the outcome demonstrates that R-RPLDN could be a surgical option for laparoscopic live donor nephrectomy. 

## Figures and Tables

**Table 1:** Reasons for selection of right RPLDN.

Right kidney (*N* = 17)	
Artery aneurysm	4
Large cyst	3
Stone	3
Artery stenosis	3
Inferior renal function	3
Angiomyolipoma	1
Left kidney (*N* = 6)	
Multiple arteries (>3)	4
Anomaly vessels	2
Other (*N* = 1)	
Left hemicolectomy	1

**Table 2:** Characteristic demographics for donors and recipients in right and left RPLDN.

	Right (*n* = 24)	Left (*n* = 509)	*P* value
Donor			
Age (yrs)	55 ± 11	54 ± 13	0.812
Sex (M/F)	6/18	174/335	0.782
Mean BMI* (kg/m^2^)	22.1 ± 2.0	22.4 ± 2.6	0.428
Renal artery (single/multiple)	20/4	386/123	0.550
Renal vein (single/multiple)	18/6	478/31	0.002
Length of renal artery (cm)	4.7 ± 0.9	3.7 ± 0.7	<0.001
Length of renal vein (cm)	1.8 ± 0.6	4.0 ± 0.8	<0.001
Recipient			
Age (yrs)	42 ± 13	38 ± 17	0.381
Sex (M/F)	12/12	331/178	0.133
HLA-identical (yes/no)	2/22	37/472	0.837
ABO incompatible (yes/no)	6/18	110/349	0.769
Preoperative DSA** (yes/no)	7/17	158/351	0.952
Immunosuppression (FK/CsA***)	24/0	474/35	0.359

*BMI: body mass index, **DSA: donor-specific HLA antibody, ***FK/CsA: tacrolimus/cyclosporine.

**Table 3:** Postoperative outcomes of right and left RPLDN.

	Right (*n* = 24)	Left (*n* = 509)	*P* value
Operating time (min)	326 ± 67	312 ± 71	0.482
Time to procurement of the kidney (min)	265 ± 66	249 ± 66	0.407
Estimated blood loss (g)	61 ± 59	51 ± 60	0.218
Warm ischemic time (min)	5.9 ± 1.9	4.8 ± 1.5	<0.001
Total ischemic time (min)	104 ± 27	100 ± 31	0.158
CO_2_ gas pressure (mmHg)	7.8 ± 1.2	7.2 ± 1.3	0.038
Postoperative hospital stay (days)	4.1 ± 1.2	3.7 ± 1.4	0.245
Slow recovery graft function* (%)	0	10 (2.0%)	0.838
Delayed graft function** (%)	0	0	—
Acute rejection (%)	5 (21%)	60 (12%)	0.315
Serum creatinine level (mg/dL)			
POD1	4.5 ± 2.4	3.7 ± 1.9	0.038
POD7	1.8 ± 1.7	1.4 ± 1.0	0.097
POD14	1.6 ± 1.7	1.5 ± 3.4	0.957
Patient survival rate			
1 year	100%	100%	0.568
5 years	100%	98.3%	
7 years	100%	97.8%	
Graft survival rate			
1 year	100%	98.2%	0.855
5 years	92.3%	95.7%	
7 years	92.3%	90.4%	

*Serum creatinine level more than 3.0 mg/dL at 4 days after transplantation. **Patients required hemodialysis after transplantation due to acute tubular necrosis. POD: postoperative days.

**Table 4:** Complications of right RPLDN and ureteral complications in recipients.

	Clavien classification	Right	Left	*P* value
*N*		24	509	
Conversion to open procedure^1^	Grade III	0	1	0.822
Slow graft function	Grade I	0	10	0.939
Intraoperative hemorrhage (≥500 g)	N/A	0	0	—
Adrenal bleeding	Grade I	0	1	0.822
Renal capsular injury	Grade I	0	1	0.822
Postoperative hemorrhage	Grade I, III^2 ^	1	6	0.767
Blood transfusion	Grade II	0	4	0.278
Lung embolism^3^	Grade II	0	1	0.822
Atelectasis	Grade I	0	1	0.822
Pneumothorax	Grade I	0	1	0.822
Subcutaneous emphysema	Grade I	1	1	0.140
Mediastinal emphysema	Grade I	0	1	0.822
Bowel complications	N/A	0	0	—
Rhabdomyolysis	N/A	0	0	—
Ureteral complications		1	4	0.513
(using double-J catheter)	Grade III	(0)	(2)	0.140
(ureter reconstruction)	Grade III	(1)	(2)	0.278
Wound infection	Grade II	1	1	0.140

Total (%)		4 (17)	33 (6.5)	0.132

^1^The reason for open donor nephrectomy was severe adhesion in the renal hilum due to previous surgery. ^2^Flank incision was added in one donor for hemostasis after left RPLDN, and blood transfusion alone in another donor. ^3^The clinical symptoms improved fortunately by conservative therapy alone.

## Abbreviations

RPLDN: Retroperitoneoscopic live donor nephrectomy
LLDN: Laparoscopic living-donor nephrectomy
WIT: Warm ischemic time
TIT: Total ischemic time
SGF: Slow recovery graft function
DGF: Delayed graft function
BMI: Body mass index.
